# Omentoplasty to Prevent Anastomotic Leak in Colorectal Anastomosis: A Systematic Review and Meta-Analysis

**DOI:** 10.7759/cureus.112138

**Published:** 2026-07-06

**Authors:** Lucas Monteiro Delgado, Bernardo Fontel Pompeu, Vitor Lauar Pimenta De Figueiredo, Eric Pasqualotto, William Silva Barbosa, Gabriel Henrique Acedo Martins, Sérgio Mazzola Poli De Figueiredo, Fernanda Bellotti Formiga

**Affiliations:** 1 Faculty of Medicine, Universidade Federal de Minas Gerais, Belo Horizonte, BRA; 2 Department of General and Colorectal Surgery, University of São Caetano do Sul, São Paulo, BRA; 3 Faculty of Medicine, Universidade de São Paulo, São Paulo, BRA; 4 Faculty of Medicine, Universidade Federal de Santa Catarina, Florianópolis, BRA; 5 Department of Surgery, University of North Carolina, Chapel Hill, USA; 6 Department of Colorectal Surgery, Santa Casa de São Paulo, São Paulo, BRA; 7 Department of Colorectal Surgery, Hospital Heliopolis, São Paulo, BRA

**Keywords:** anastomotic leakage, colorectal anastomosis, colorectal surgery, meta-analysis, omentoplasty, postoperative complications, surgical revision, systematic review

## Abstract

Anastomotic leakage (AL) increases morbidity and mortality in colonic and rectal surgery. Omentoplasty (OMP), the use of a pedicled flap from the omentum to shield the anastomotic site, has been proposed as a potential method to prevent this complication. This study aims to evaluate the effectiveness of OMP in reducing the incidence of AL and its clinical repercussions. We searched PubMed, Scopus, and the Cochrane Library for randomized controlled trials (RCTs) and observational studies comparing OMP and non-omentoplasty (n-OMP) in colonic and rectal surgeries. Mean differences (MDs) were computed for continuous outcomes and odds ratios (ORs) for binary endpoints, with 95% confidence intervals (CIs). Heterogeneity was assessed using I² statistics. Statistical analysis was performed using R software, version 4.3.3 (R Foundation for Statistical Computing, Vienna, Austria). Eight studies (four RCTs and four observational studies) involving a total of 5,330 patients were included, with 806 patients (15%) in the OMP group and 4,524 patients (85%) in the n-OMP group. Clinical AL, reported in three studies that distinguished leak type, was significantly reduced in the OMP group (OR 0.35; 95% CI 0.15-0.81; p=0.01; I²=0). There were no significant differences in overall AL (OR 0.61; 95% CI 0.33-1.12; p=0.11; I²=56%), radiological AL (OR 0.77; 95% CI 0.40-1.47; p=0.42; I²=0%), reoperation rates (OR 0.63; 95% CI 0.37-1.08; p=0.09; I²=0%), mortality (OR 0.78; 95% CI 0.33-1.86; p=0.58; I²=25%), or postoperative infection rates.

OMP was not associated with a significant reduction in overall or radiological AL. Clinical AL, however, was significantly lower in the OMP group, a finding drawn from a small subset of studies that may point to a role for OMP in containing the consequences of a leak rather than preventing it outright. No significant differences were observed in reoperation rates, mortality, or postoperative infection rates. Given the heterogeneity and inclusion of observational data, further RCTs are needed to confirm these findings.

## Introduction and background

A colorectal anastomosis is the surgical reconnection of the bowel after resection, restoring intestinal continuity between the remaining ends. Its failure to heal, whether from poor perfusion, tension on the suture or staple line, contamination, or technical factors, allows luminal contents to escape into the peritoneal cavity and defines anastomotic leakage (AL). AL is classified as overall, encompassing all reported leaks regardless of detection method; clinical, when accompanied by signs such as peritonitis, fecal or purulent drainage, or sepsis prompting intervention; or radiological, when identified on imaging in the absence of clinical signs. AL is a major concern in colorectal surgery due to its association with increased morbidity and mortality rates [1]. It is considered the most frequent major surgical complication following colorectal surgery, with reported incidence ranging from 2% to 14% [2]. Patients with AL require surgical revision in approximately 10% to 35% of cases [1].

Omentoplasty (OMP) has been proposed as a technique to reduce AL. This surgical procedure involves using a pedicled flap from the omentum to cover or wrap the anastomotic site. Beyond the mechanical protection it provides, the omentum is a richly vascularized and immunologically active tissue: it contains milky spots rich in macrophages and lymphoid aggregates that clear bacteria and particulate matter from the peritoneal cavity, and it releases angiogenic and growth factors that promote granulation tissue formation and neovascularization at the anastomotic site [3]. These properties give it a theoretical dual role, limiting bacterial contamination while also localizing and dampening the peritoneal inflammatory response. This raises a distinction worth stating explicitly: OMP may act less by preventing a leak from occurring and more by containing its spread and moderating its clinical consequences once a leak occurs, a distinction we revisit when interpreting our findings on clinical versus radiological leakage. Several systematic reviews and meta-analyses have suggested potential benefits of OMP in reducing AL in other gastrointestinal and esophageal procedures [4-6]. Grigor et al. found that omental wrapping was among the few interventions associated with reduced leak rates after esophagectomy, though the certainty of evidence was low given the small number of trials [4]. Similarly, Wiggins et al. reported a modest protective effect of OMP across mixed gastrointestinal anastomoses but noted substantial heterogeneity and a predominance of observational data [5].

However, the evidence supporting the routine use of OMP in colorectal surgery remains inconclusive. The most recent meta-analysis on this topic, by Sahebally et al., pooled four randomized clinical trials (RCTs) totaling 1,067 patients and concluded that OMP significantly reduced AL [7]. This conclusion rested on a comparatively small sample, and two of the four included trials carried bias concerns that may have favored OMP, leaving the true effect uncertain. To address this gap and as a methodological contribution over prior reviews restricted to RCTs alone, we conducted a comprehensive literature search incorporating both RCTs and observational studies [8-15]. This broader inclusion increases the available sample size and allows analysis of additional outcomes beyond what RCT-only evidence supports, but it also introduces a tradeoff: observational data carry a higher risk of confounding than randomized evidence. Therefore, we performed an updated systematic review and meta-analysis, combining randomized and observational evidence, to compare the outcomes of OMP versus non-omentoplasty (non-OMP) in patients undergoing colorectal surgery.

## Review

Methods

Protocol and Registration

We performed this systematic review and meta-analysis according to the Cochrane Handbook for Systematic Reviews of Interventions and structured it according to the Preferred Reporting Items for Systematic Reviews and Meta-Analysis (PRISMA) guidelines [16-17]. The study protocol was registered in the International Prospective Register of Systematic Reviews (PROSPERO) under registration number CRD42024552817 [18].

Outcomes

The outcomes of interest were: (1) AL, further classified as overall, clinical, or radiological; (2) reoperation; (3) mortality; (4) mortality due to abdominal complications; (5) postoperative infection, including superficial surgical site infection (SSI), deep SSI, and intra-abdominal infection, phlegmon, or abscess; (6) operative blood loss; (7) length of hospital stay; and (8) operative time. Overall, AL comprised all reported leaks regardless of detection method. Clinical AL was defined as a leak associated with clinical signs or symptoms, such as peritonitis, fecal or purulent drainage, or sepsis, prompting further intervention. Radiological AL was defined as a leak identified on imaging, such as contrast enema or CT, in the absence of corresponding clinical signs.

Eligibility Criteria

Inclusion in this meta-analysis was restricted to studies that met all the following eligibility criteria: (1) RCTs or observational studies; (2) comparison of ileoileal, ileocolic, colocolic, ileorectal, and colorectal anastomoses with OMP versus anastomoses without OMP; (3) enrollment of adult patients aged ≥18 years; and (4) reporting at least one outcome of interest. Studies meeting any of the following criteria were excluded: (1) non-colorectal surgeries; (2) colorectal surgeries that did not require anastomosis; (3) studies lacking a control group or with an inappropriate control group; (4) unsuitable publication types, including case reports, conference abstracts, meta-analyses, reviews, and animal experiments; and (5) inability to obtain original data from the corresponding author. Detailed inclusion and exclusion criteria for each study are provided in Table 1.

**Table 1:** Inclusion and exclusion criteria for the included studies. ACS-NSQIP: American College of Surgeons National Surgical Quality Improvement Program; ASA: American Society of Anesthesiologists.

Study	Inclusion Criteria	Exclusion Criteria
Nasiri et al., 2017 [14]	Patients scheduled for elective segmental resection of the small intestine and colon or colectomy requiring primary anastomosis. Any age or gender.	History of chemotherapy or radiotherapy; Conditions like anemia or hypoproteinemia; Prior intestinal surgeries; Signs of malnutrition.
Tocchi et al., 2000 [15]	Adult patients with rectal adenocarcinoma scheduled for anterior resection.	Incomplete staple rings post-anastomosis; Omentum unsuitable due to atrophy or scarring from previous surgeries.
Agnifili et al., 2004 [12]	Individuals with carcinoma, benign tumors, diverticular disease, or other colon/rectum conditions, requiring open surgical resection.	Patients requiring laparoscopic approaches; Conditions making omentoplasty infeasible, like insufficient omental size.
Ali et al., 2024 [8]	Surgical intervention for malignancy, benign tumors, and diverticular disease with anastomosis below the peritoneal reflection. Elective or emergency surgery.	Laparoscopic procedures: Omentoplasty is infeasible due to the absence or unsuitability of the omentum.
Merad et al., 1998 [13]	Conditions like carcinoma to benign tumors, Crohn's disease affecting the right colon to mid-rectum, requiring resection and immediate reconstruction.	Emergency surgeries.
Ozben et al., 2016 [9]	Segmental colectomy with low pelvic anastomosis from the ACS-NSQIP database. Patients included from multiple centers.	Emergency procedures; ASA score > 4; Absence of omentum due to prior surgical removals.
Ozben et al., 2018 [10]	Similar to Ozben 2016: Patients undergoing low pelvic anastomosis, detailed data from ACS-NSQIP 2012 targeted colectomy file.	Emergency cases; ASA score above 4; Lack of omentum due to surgeries or congenital absence.

Search Strategy and Study Selection

We systematically searched PubMed, Scopus, and the Cochrane Library databases from inception to July 1, 2024. The search strategy was: ("Rectal Cancer Surgery" OR "Colorectal Surgery" OR "Intestinal Resection" OR "Colorectal Resection" OR "Colon Surgery" OR "Rectal Resection" OR "Low Anterior Resection" OR "Anastomosis Leak") AND ("Omentoplasty" OR "Omental Pedicled Flap" OR "Omental Pedicle Flap" OR "Epiploplasty" OR "Colorectal Anastomosis" OR "Rectal Anastomosis" OR "Colon Anastomosis"). We also reviewed the references of the included studies and previous systematic reviews and meta-analyses to identify additional studies [19].

Two authors (L.M.D. and V.L.P.F.) independently conducted the search, imported the results into Rayyan Software (Qatar Computing Research Institute, Qatar Foundation, Doha, Qatar), and screened the studies. After excluding duplicates and titles/abstracts unrelated to the clinical question, the eligibility of each remaining study was assessed through full-text review. Disagreements were resolved by a third author (B.F.P.).

Data Extraction

Two authors (L.M.D. and W.S.B.) extracted data from the included studies. Extracted data included title, authors, year of publication, study design, country of origin, number of participants, patient characteristics, surgical procedures and their indications, type of anastomosis, air-tightness test, and reported outcomes.

Risk of Bias and Certainty Assessment

Non-randomized studies were assessed using the Risk Of Bias In Non-randomized Studies of Interventions (ROBINS-I) tool [20], whereas RCTs were evaluated using the Cochrane Risk of Bias 2 (RoB 2) tool [21]. Two independent authors (L.M.D. and V.L.P.F.) conducted the risk of bias assessment, and disagreements were resolved by consensus with the senior author (B.F.P.). Publication bias could not be adequately assessed because tests for funnel plot asymmetry have insufficient power to distinguish chance from true asymmetry when fewer than ten studies are included [22]. The certainty of evidence for each outcome was assessed using the Grading of Recommendations Assessment, Development and Evaluation (GRADE) approach, considering risk of bias, inconsistency, indirectness, imprecision, and publication bias. Certainty was independently rated by two authors as high, moderate, low, or very low, with disagreements resolved by consensus.

Statistical Analysis

Mean differences (MDs) and odds ratios (ORs) with 95% confidence intervals (CIs) were pooled for continuous and binary outcomes, respectively. A P-value of <0.05 was considered statistically significant for overall effect estimates. DerSimonian and Laird random-effects models were used for all outcomes [23]. Heterogeneity was assessed using the Cochran Q test and I² statistics; P-values <0.10 and I² >25% were considered indicative of significant heterogeneity. We performed a leave-one-out sensitivity analysis for outcomes with significant heterogeneity or disproportionate study weight. Statistical analyses were performed using R statistical software, version 4.3.3 (R Foundation for Statistical Computing, Vienna, Austria).

Data Availability

The data extracted from included studies and the R scripts used for the meta-analysis are available from the corresponding author upon reasonable request.

Results

Study Selection and Characteristics

**Figure 1:**
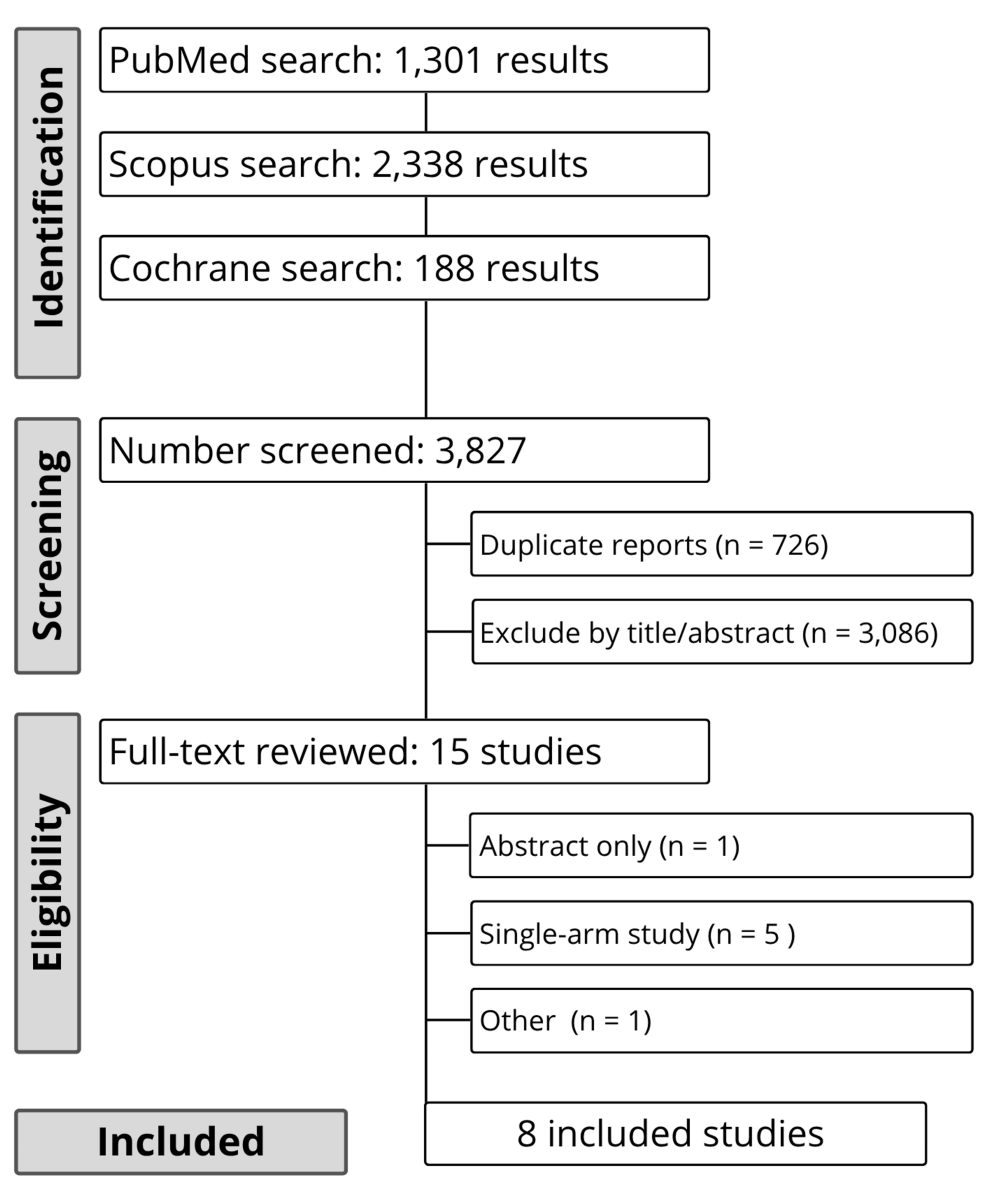
Figure 1. PRISMA flow diagram of study selection. PRISMA: Preferred Reporting Items for Systematic Reviews and Meta-Analyses.

As detailed in Figure 1, the initial search yielded 3,827 studies. After duplicate removal and title and abstract screening, 15 full-text studies met the prespecified eligibility criteria and were assessed in detail. Ultimately, eight studies were included in the meta-analysis, consisting of four RCTs and four observational studies [8-15], with a total of 5,330 patients. Among these, 806 patients (15.1%) underwent OMP, whereas 4,524 patients (84.9%) did not.

The eight included studies were conducted across six countries: France, Iran, Italy, the Netherlands, Pakistan, and the United States. Individual study sizes ranged from 51 patients in Klaver et al. [11] to 2,891 patients in Ozben et al. [10]. Surgical indications were predominantly colorectal malignancy, with a smaller proportion of benign conditions, including diverticular disease, inflammatory bowel disease, and intestinal obstruction or perforation.

Mean patient age ranged from 51.8 to 66.6 years across studies. The proportion of male patients was broadly balanced between the OMP and n-OMP groups overall, comprising 52.9% and 54.0%, respectively. Reporting of operative approach, including open, laparoscopic, or robotic surgery, anastomotic technique, including stapled or hand-sewn anastomosis, and intraoperative air leak testing was incomplete across several studies.

Anastomotic location and type also varied across the included studies. Six studies, including Agnifili et al. [12], Ali et al. [8], Klaver et al. [11], Ozben et al. [9-10], and Tocchi et al. [15], specifically evaluated colorectal anastomoses, reflecting predominantly left-sided or low pelvic resections. Merad et al. [13] included a broader anatomic range, encompassing ileocolic, colorectal, and ileorectal anastomoses, whereas Nasiri et al. [14] evaluated exclusively proximal anastomoses, including ileoileal, ileocolic, and colocolic anastomoses, with no rectal or low pelvic component. This anatomic heterogeneity included anastomoses with substantially different baseline risks of leakage. The baseline characteristics of the study populations are detailed in Table 2.

**Table 2:** Baseline characteristics of the included studies. NA: not available; OMP: omentoplasty; n-OMP: non-omentoplasty; RCT: randomized controlled trial. ^†^The remaining data were missing or categorized as other.

First author, year	Design	Country	No. of patients OMP/n-OMP	Disease	Topography of the anastomosis	Mean age, years OMP/n-OMP	Male, n OMP/n-OMP	Operative approach, %		Anastomosis method, %		Operative air leak test, %		
								Open	Laparoscopic/Robotic	Stapled	Handsewn	Negative	Positive	Not performed
Agnifili et al., 2004 [12]	Prospective RCT	Italy	62/64	Malignancy, benign tumor, diverticular disease, and others.	Colorectal anastomosis	64/61	30/25	100	0	100	0	-	-	100
Ali et al., 2024 [8]	Retrospective cohort	Pakistan	106/747	Malignancies, benign tumors, diverticular disease	Colorectal anastomosis	58.9/58.5	69/459	23.4	52.6†	83.7	8.6†	59.1	3.9	27.5†
Klaver et al., 2008 [11]	Retrospective cohort	The Netherlands	31/20	Rectal cancer	Colorectal anastomosis and APR	62.8/66.6	15/10	NA	NA	NA	NA	NA	NA	NA
Merad et al., 1998 [13]	Prospective RCT	France	341/364	Carcinoma, benign tumors, colonic Crohn’s disease, diverticular disease	Ileocolic, colorectal and ileorectal anastomosis	66/66	158/189	NA	NA	41.7	58.3	73.3	6	20.7
Nasiri et al., 2017 [14]	Prospective RCT	Iran	62/62	Malignancy, intestinal obstruction or perforation, ileostomy closure, colon inertia and mesenteric ischemia	Ileoileal, ileocolic and colocolic anastomosis	51.8/54.1	46/31	100	0	NA	NA	NA	NA	NA
Ozben et al., 2016 [9]	Prospective cohort	United States	65/403	Rectal cancer	Colorectal anastomosis	59.2/59.6	40/286	67.5	32.5	88.5	11.5	NA	NA	NA
Ozben et al., 2018 [10]	Retrospective cohort	United States	86/2805	Colorectal cancer, diverticulitis, Inflammatory bowel diseases and others	Colorectal anastomosis	60.0/60.2	36/1411	32.4	67.6	NA	NA	NA	NA	NA
Tocchi et al., 2000 [15]	Prospective RCT	Italy	53/59	Adenocarcinoma of the rectum	Colorectal anastomosis	64.3/65.2	32/32	100	0	100	0	NA	NA	NA

Anastomotic Leakage

**Figure 2:**
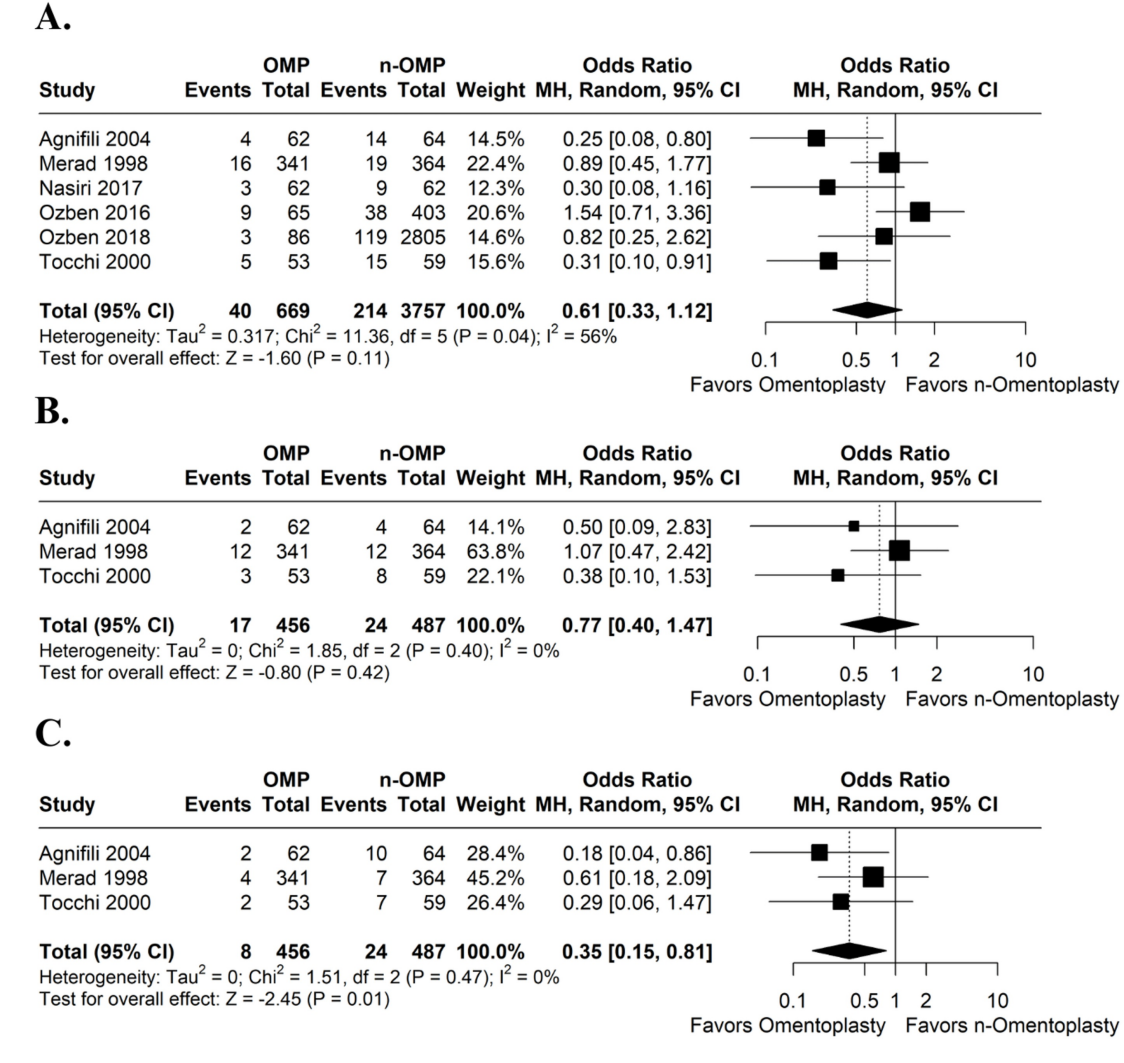
Forest plots for anastomotic leakage (AL) outcomes, omentoplasty (OMP) versus non-omentoplasty (n-OMP) (A) Overall AL; (B) Radiological AL; (C) Clinical AL Data are presented as odds ratios (OR) with 95% confidence intervals (CI), pooled under a DerSimonian and Laird random-effects model. References: [9,10,12-15]

There were no significant differences between the OMP and n-OMP groups in terms of overall AL (40/669 vs 214/3757 events; OR 0.61; 95% CI 0.33-1.12; p=0.11; I²=56%; Figure 2A) or radiological AL (17/456 vs 24/487 events; OR 0.77; 95% CI 0.40-1.47; p=0.42; I²=0%; Figure 2B). However, clinical AL, reported by the three studies that distinguished clinical from subclinical leaks [12-13,15], was significantly lower in the OMP group (8/456 vs 24/487 events; OR 0.35; 95% CI 0.15-0.81; p=0.01; I²=0%; Figure 2C).

Reoperation, Mortality, and Mortality Due to Abdominal Complications

**Figure 3:**
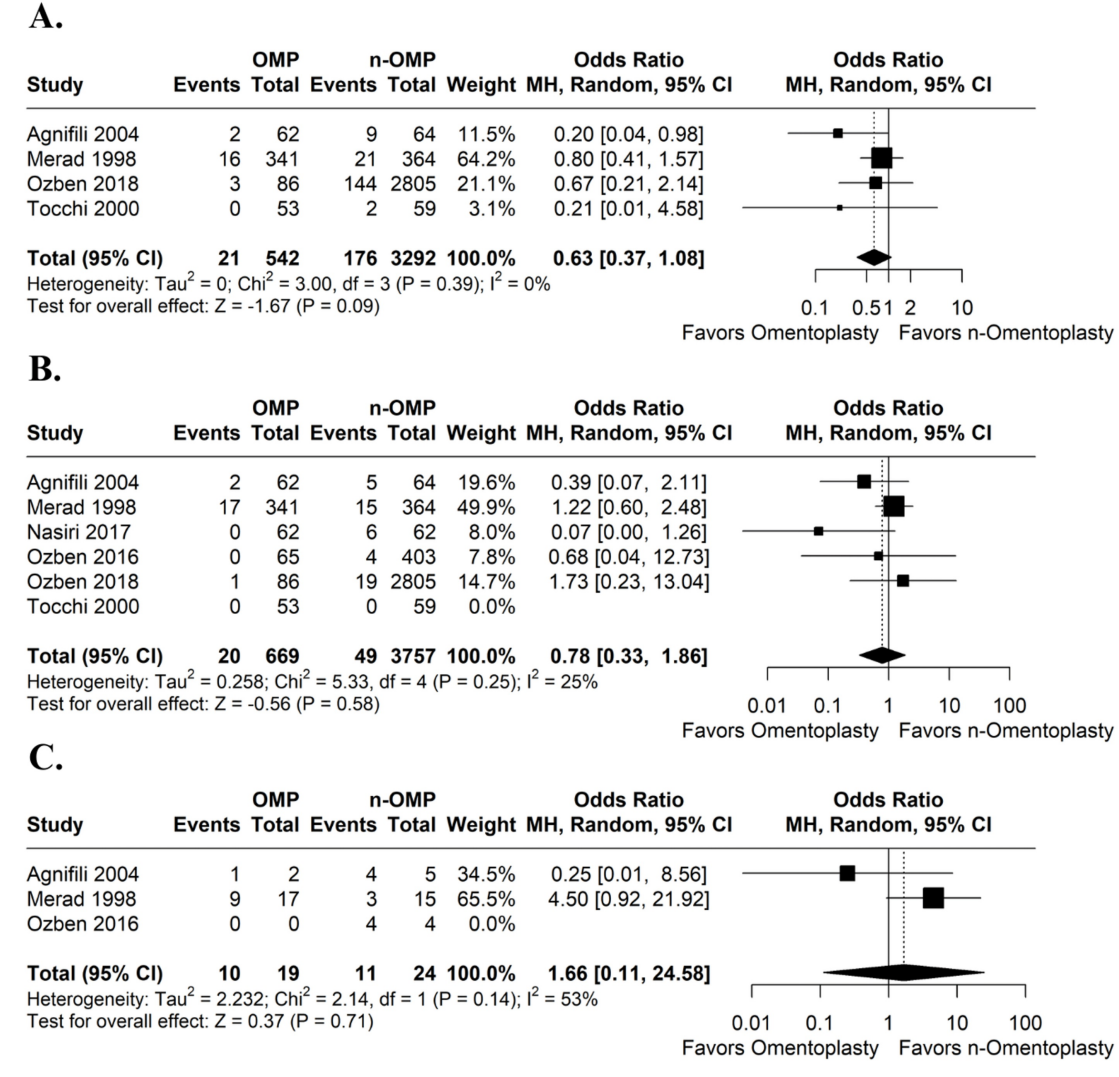
Forest plots for reoperation and mortality outcomes, omentoplasty (OMP) versus non-omentoplasty (n-OMP) (A) Reoperation; (B) Overall mortality; (C) Mortality due to abdominal complications Data are presented as odds ratios (OR) with 95% confidence intervals (CI), pooled under a DerSimonian and Laird random-effects model. References: [9,10,12-15]

There were no significant differences between the OMP and n-OMP groups in reoperation rates (21/542 vs 176/3292 events; OR 0.63; 95% CI 0.37-1.08; p=0.09; I²=0%; Figure 3A), overall mortality (20/669 vs 49/3757 events; OR 0.78; 95% CI 0.33-1.86; p=0.58; I²=25%; Figure 3B), or mortality due to abdominal complications (10/19 vs 11/24 events; OR 1.66; 95% CI 0.11-24.58; p=0.71; I²=53%; Figure 3C). The mortality-due-to-abdominal-complications estimate is based on a subset of the overall mortality events reported by the three studies with cause-of-death data: 10 of 19 deaths in the OMP arm and 11 of 24 deaths in the n-OMP arm were attributed to abdominal complications. This small number of contributing deaths, rather than patients, should be kept in mind when interpreting the wide confidence interval.

Postoperative Infection, Operative Blood Loss, Length of Hospital Stay, and Operative Time

**Figure 4:**
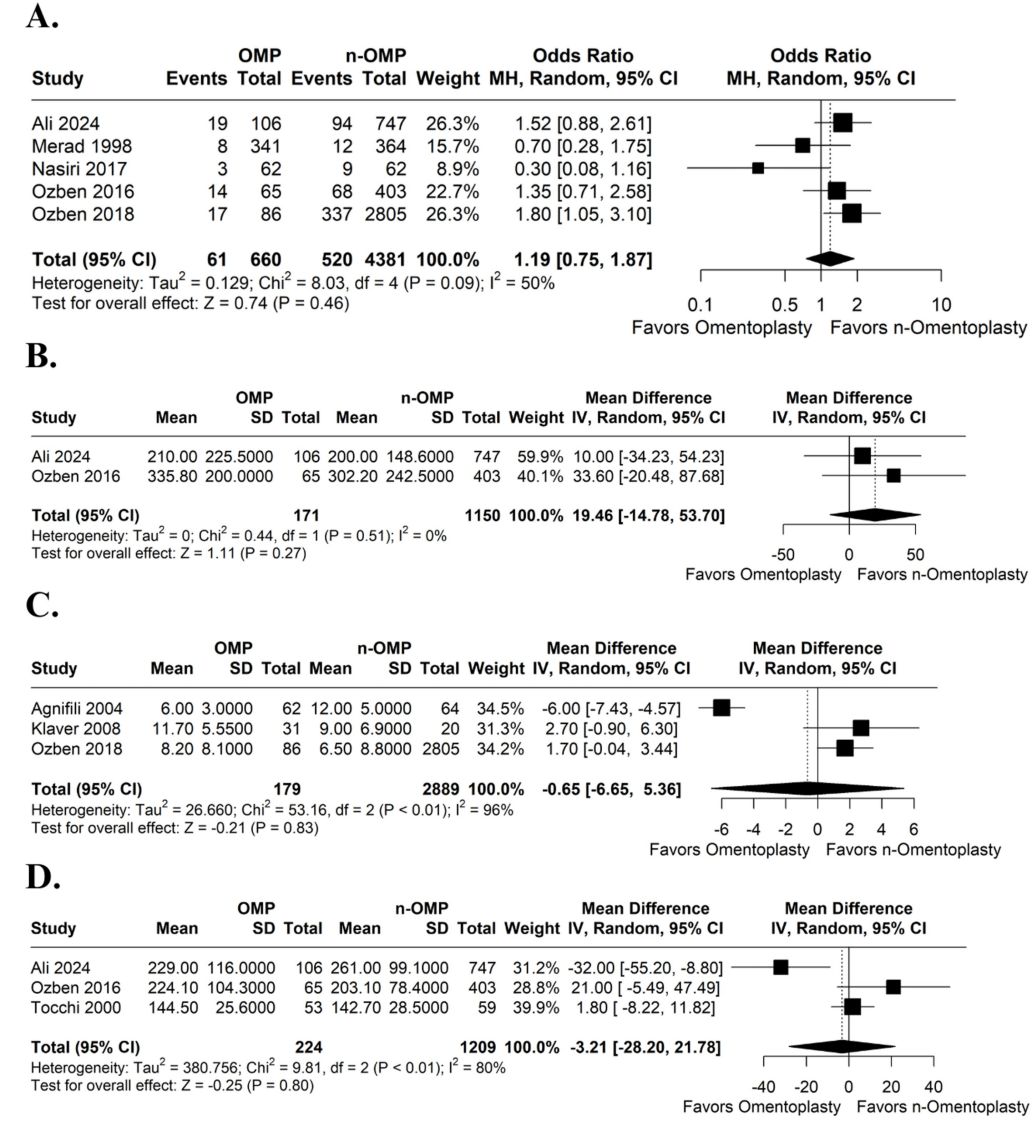
Forest plots for postoperative infection and perioperative outcomes, omentoplasty (OMP) versus non-omentoplasty (n-OMP) (A) Postoperative infection, presented as odds ratios (OR); (B) Operative blood loss (mL); (C) length of hospital stay (days); and (D) operative time (minutes), presented as mean differences (MD). Data are pooled under a DerSimonian and Laird random-effects model. References: [8-15]

There were no significant differences between the OMP and n-OMP groups in postoperative infection rates (61/660 vs 520/4381 events; OR 1.19; 95% CI 0.75-1.87; p=0.46; I²=50%; Figure 4A), operative blood loss (MD (mL) 19.46; 95% CI -14.78-53.70; p=0.27; I²=0%; n=171 vs 1150; Figure 4B), length of hospital stay (MD (days) -0.65; 95% CI -6.65-5.36; p=0.83; I²=96%; n=179 vs 2889; Figure 4C), and operative time (MD (minutes) -3.21; 95% CI -28.20-21.78; p=0.80; I²=80%; n=224 vs 1209; Figure 4D). Given the extreme heterogeneity observed for length of hospital stay (I²=96%) and operative time (I²=80%), these pooled MDs do not represent a common underlying effect and should be interpreted with caution rather than as summary estimates of a consistent treatment effect.

Sensitivity Analysis

**Figure 5:**
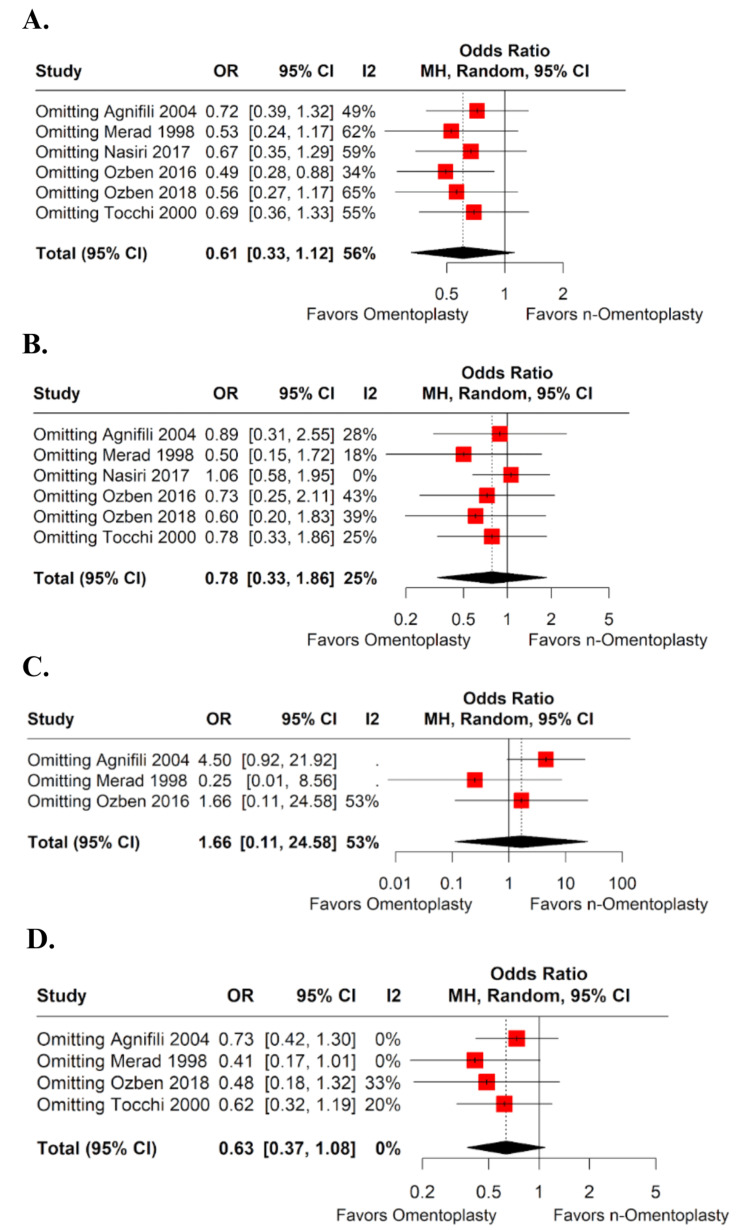
Leave-one-out sensitivity analysis for anastomotic leakage, mortality, and reoperation outcomes, omentoplasty (OMP) versus non-omentoplasty (n-OMP). (A) Overall anastomotic leakage; (B) Overall mortality; (C) Mortality due to abdominal complications; (D) Reoperation. Each row shows the pooled odds ratio (OR) with 95% confidence interval (CI) after sequential exclusion of the named study, under a DerSimonian and Laird random-effects model. I²: heterogeneity statistic References: [9,10,12-15]

**Figure 6:**
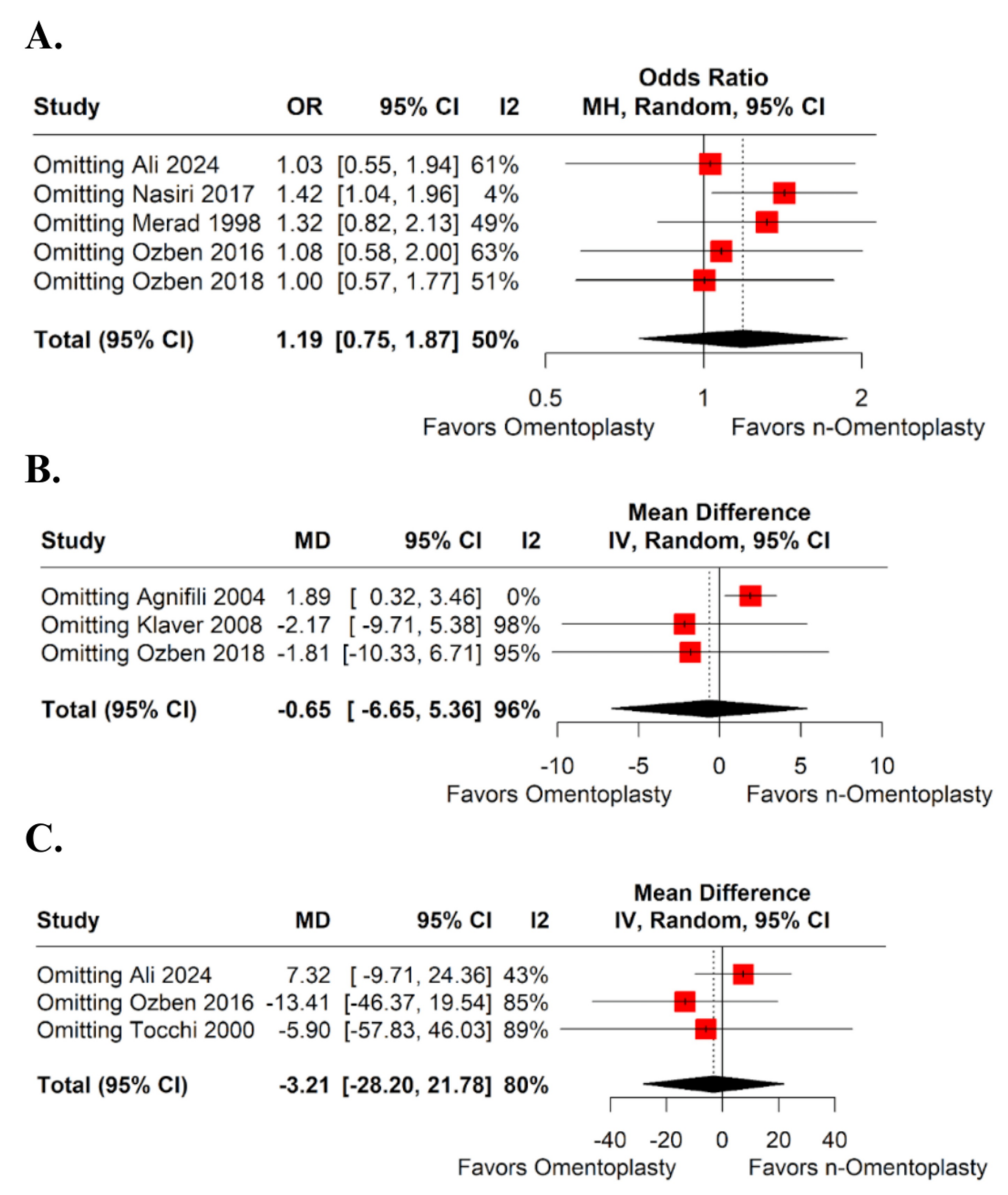
Leave-one-out sensitivity analysis for postoperative infection, length of hospital stay, and operative time, omentoplasty (OMP) versus non-omentoplasty (n-OMP). (A) Postoperative infection, presented as odds ratios (OR); (B) Length of hospital stay (days); and (C) operative time (minutes), presented as MDs. Each row shows the pooled estimate after sequential exclusion of the named study, under a DerSimonian and Laird random-effects model. MD: mean difference; CI: confidence interval; I²: heterogeneity statistic References: [9-15]

The leave-one-out sensitivity analysis was conducted to evaluate the impact of individual studies on the pooled effect sizes and heterogeneity across outcomes. The removal of Ozben et al. [9] shifted the AL outcome to statistical significance in favor of OMP (OR 0.49; 95% CI 0.28-0.88; I²=34%). For the length of hospital stay outcome, the removal of Agnifili et al. [12] reversed the direction of effect and reached significance in favor of the n-OMP group (MD 1.89; 95% CI 0.32-3.46; I²=0%). Similarly, for postoperative infection, the removal of Nasiri et al. [14] reversed the direction of effect and reached significance in favor of the n-OMP group (OR 1.42; 95% CI 1.04-1.96; I²=4%). For reoperation, the removal of Merad et al. [13], which contributed the largest weight (64.8%) to this outcome, shifted the pooled estimate closer to statistical significance in favor of OMP (OR 0.41; 95% CI 0.17-1.01; I²=0%), although the interval still marginally included the null value. No major changes in significance were observed with the removal of individual studies for the other outcomes. The leave-one-out sensitivity analysis plots are provided in Figures 5-6.

Risk of Bias and Certainty Assessment

**Figure 7:**
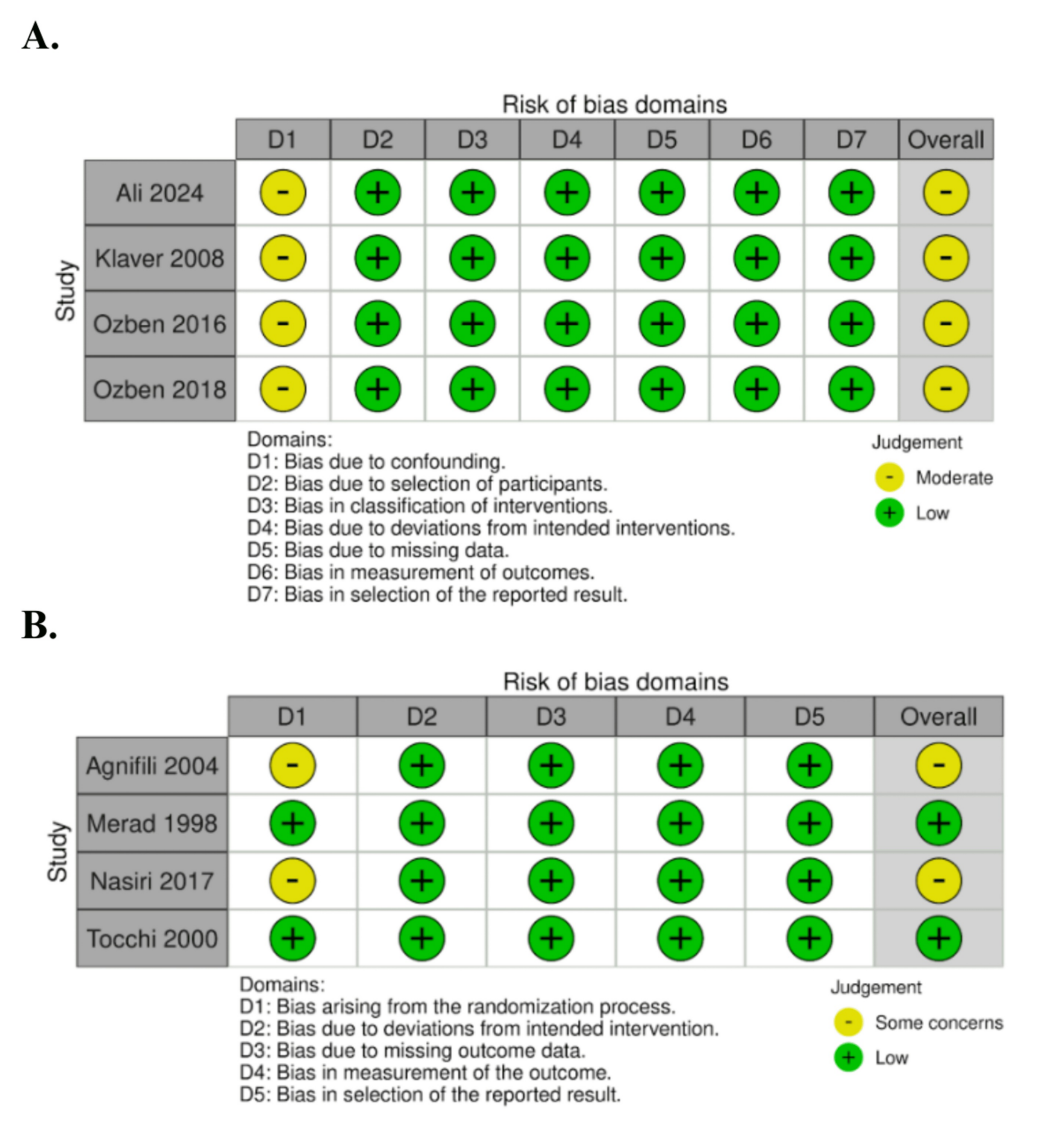
Risk of bias assessment for included studies (A) Risk of Bias in Non-randomized Studies of Interventions (ROBINS-I) [15] for the four observational studies; (B) Cochrane risk-of-bias tool for randomized trials (RoB 2) [16] for the four randomized controlled trials (RCTs). Domains assessed are tool-specific and defined beneath each panel. Green (+) indicates low risk of bias; yellow (-) indicates moderate risk of bias (ROBINS-I) or some concerns (RoB 2). References: [8-15]

The appraisal of studies is detailed in Figure 7. Two RCTs included in this meta-analysis [12,14] were identified with some concerns of bias, primarily due to baseline imbalances related to the disease requiring surgery between the OMP and n-OMP groups. The remaining RCTs [13,15] were classified as having a low risk of bias. All observational studies were assessed as having a moderate risk of bias, mainly due to confounding factors [8-11].

The certainty of evidence, assessed using the GRADE approach, was low for most outcomes, including overall AL, clinical AL, reoperation, overall mortality, and postoperative infection, reflecting the combination of randomized and observational evidence, risk of bias, inconsistency, or imprecision from limited event counts. Radiological AL was the only outcome rated with moderate certainty. Certainty was very low for mortality due to abdominal complications and for the three continuous outcomes (operative blood loss, length of hospital stay, and operative time), reflecting small sample sizes and, for length of stay and operative time, very high statistical heterogeneity (I²=96% and 80%, respectively), precluding interpretation of the pooled estimate as a common effect. The complete domain-by-domain assessment is presented in Table 3.

**Table 3:** Grading of Recommendations Assessment, Development and Evaluation (GRADE) summary of the findings. a. Downgraded for risk of bias: mixed randomised controlled trial (RCT) and observational evidence; two out of four RCTs had "some concerns" (Risk of Bias 2 (RoB 2)), and the observational studies were rated as having "moderate" risk of bias using the Risk Of Bias In Non-randomised Studies - of Interventions (ROBINS-I) tool, primarily due to confounding; b. Downgraded for inconsistency (I² = 56%); the direction of effect shifted following leave-one-out removal of Ozben et al. [10]; c. Downgraded for imprecision: only 41 events across three studies; d. Downgraded by two levels for imprecision: statistically significant result, but only 32 total events across three studies, representing a signal from a limited evidence base; e. Downgraded for risk of bias/indirectness: dominated by Merad et al. [13] (64.8% weight), in which 70% of reoperations were unrelated to anastomotic leak (AL); f. Downgraded for imprecision: wide confidence interval (CI) relative to a low event rate; g. Denominator represents the total number of deaths across the contributing studies, not the total number of enrolled patients; h. Downgraded to the lowest certainty level for very serious imprecision (CI spans >200-fold; only 19 and 24 deaths), together with inconsistency and risk of bias; i. Downgraded for very serious imprecision: two studies, n = 321, with the CI crossing the null substantially; j. Negative values favour omentoplasty (OMP); k. Downgraded by two levels for very serious inconsistency (I² = 96% and 80%); the pooled estimate does not represent a common effect.

Outcome	Number of participants (studies)	Risk with n-OMP	Risk with OMP	Absolute effect (95% CI)	Relative effect (95% CI)	Certainty
Overall anastomotic leakage (AL)	4,426 (6 studies)	57 per 1,000	36 per 1,000	21 fewer per 1,000 (37 fewer to 6 more)	OR 0.61 (0.33–1.12)	⊕⊕◯◯ Lowᵃᵇ
Radiological AL	943 (3 RCTs)	49 per 1,000	38 per 1,000	11 fewer per 1,000 (29 fewer to 22 more)	OR 0.77 (0.40–1.47)	⊕⊕⊕◯ Moderateᶜ
Clinical AL	943 (3 RCTs)	49 per 1,000	18 per 1,000	32 fewer per 1,000 (42 fewer to 9 fewer)	OR 0.35 (0.15–0.81)	⊕⊕◯◯ Lowᵈ
Reoperation	3,834 (4 studies)	54 per 1,000	34 per 1,000	19 fewer per 1,000 (33 fewer to 4 more)	OR 0.63 (0.37–1.08)	⊕⊕◯◯ Lowᵉ
Mortality (overall)	4,426 (6 studies)	13 per 1,000	10 per 1,000	3 fewer per 1,000 (9 fewer to 11 more)	OR 0.78 (0.33–1.86)	⊕⊕◯◯ Lowᵇᶠ
Mortality due to abdominal complications	43 deaths (3 studies)ᵍ	—	—	not estimable at population level	OR 1.66 (0.11–24.58)	⊕◯◯◯ Very Lowʰ
Postoperative infection	5,041 (5 studies)	119 per 1,000	138 per 1,000	19 more per 1,000 (27 fewer to 83 more)	OR 1.19 (0.75–1.87)	⊕⊕◯◯ Lowᵃᶠ
Operative blood loss	1,321 (2 obs. studies)	—	—	MD 19.46 mL higher (14.78 lower to 53.70 higher)	—	⊕◯◯◯ Very Lowⁱ
Length of hospital stay	3,068 (3 studies)	—	—	MD 0.65 days lower (6.65 lower to 5.36 higher)ʲ	—	⊕◯◯◯ Very Lowᵏ
Operative time	1,433 (3 studies)	—	—	MD 3.21 min lower (28.20 lower to 21.78 higher)ʲ	—	⊕◯◯◯ Very Lowᵏ

Discussion

In this systematic review and meta-analysis of eight studies comprising 5,330 patients, we evaluated the impact of OMP in colorectal anastomosis. Our key findings were as follows: (1) OMP did not significantly reduce AL compared to n-OMP; and (2) reoperation rates, mortality, postoperative infection rates, intraoperative blood loss, length of hospital stay, and operative times were similar between the groups.

Reducing AL remains a major concern in colorectal surgery, as it is associated with increased morbidity, mortality, the need for permanent stoma formation, and even cancer recurrence [24]. In our analysis, OMP reinforcement in colorectal anastomosis did not lead to a significant difference in overall leakage rates between groups. A limitation of this analysis was the inability to distinguish between clinical and subclinical leaks in most studies and whether they resulted in clinical repercussions or reoperations; only three studies differentiated leaks by detection method [12-13,15]. Our analysis showed no difference between groups in radiological AL, while clinical AL rates were lower in the OMP group. Variations in anastomotic technique, including stapled versus hand-sewn anastomoses, may have contributed to the overall variability in the results.

Sahebally et al. conducted the most recent meta-analysis on this topic [7], incorporating data from four RCTs with a total of 1,067 patients. Their analysis concluded that OMP significantly reduced AL compared to n-OMP in colorectal anastomosis. However, these results differ from our findings and may be explained by the small sample size of the previous meta-analysis and the bias concerns present in two of the included studies, which could have skewed the outcomes in favor of OMP. Additionally, the substantial heterogeneity in the data from that study limits the ability to draw definitive conclusions on this subject. These discrepancies reinforce the need for well-conducted RCTs to obtain more reliable evidence on the role of OMP in colonic and rectal surgery.

OMP has also been recommended for managing conditions such as hepatic cystic echinococcosis and esophageal surgeries [5,25]. However, this technique exhibits significant technical variations in the literature. Some studies describe simple placement of the omentum over the anastomosis without fixation, while others detail specific suturing or stapling techniques to secure the flap in place. The extent of anastomotic coverage, whether the omentum is wrapped circumferentially or draped over a single surface, and the length and vascular pedicle of the flap used are similarly inconsistent across studies. This technical heterogeneity, largely unreported in the included studies, likely contributes to variability in the observed effect of OMP and limits the ability to identify an optimal technique.

Anastomoses in the left colon generally have a higher risk of AL compared to ileocolic anastomoses, due to factors such as poorer blood supply, higher bacterial load, and technical challenges associated with low pelvic anastomosis [26]. Six of the eight included studies evaluated colorectal anastomoses specifically, reflecting predominantly left-sided or low pelvic resections, while Merad et al. [13] included a broader anatomic range spanning ileocolic to ileorectal anastomoses, and Nasiri et al. [14] evaluated exclusively proximal anastomoses with no rectal or low pelvic component. Pooling anastomoses across this range of baseline leak risk may have diluted a protective effect of OMP that is more pronounced in high-risk pelvic anastomoses, where the theoretical benefits of mechanical support and containment are likely greatest. Future trials should specifically evaluate the role of OMP in low rectal and pelvic anastomoses, where both the incidence and consequences of AL are highest.

The dissociation we observed between clinical AL, which was reduced in the OMP group, and overall and radiological AL, which were not, suggests that OMP may not primarily prevent an anastomosis from leaking, but rather limit the contamination and sepsis that follow once a leak has occurred. In addition to potentially minimizing AL, OMP has been suggested to reduce the clinical impact of an AL by theoretically blocking its intraperitoneal spread [3]. This containment effect may help mitigate more severe clinical consequences [6]. A diverting stoma may act through a related but distinct pathway: by diverting the fecal stream away from the anastomosis, it can reduce contamination at the leak site and mask the clinical signs of a leak that is nonetheless detectable radiologically, potentially contributing to the dissociation we observed between clinical and radiological AL rates. Predictive risk factors for leaks include a positive air tightness test and, in the case of stapled anastomosis, stapled ring disruption, both of which can influence the occurrence of AL regardless of whether OMP is performed [27-28]. Therefore, despite the lack of significant differences in overall AL rates between groups, the reduced rates of clinical AL in our analysis support a potential protective effect of OMP in preventing severe repercussions of leaks.

This distinction carries meaningful clinical weight. AL, and clinically apparent leakage in particular, is associated with prolonged hospital stay, a higher rate of surgical reintervention, and increased mortality [29]. Beyond the acute episode, patients who develop AL after colorectal cancer surgery report impaired quality of life, an effect most pronounced within the first postoperative year [30]. The broader consequences of AL extend to permanent stoma formation and poorer oncological outcomes, and its downstream burden is not limited to the index hospitalization [31]. A reduction in clinically apparent leaks, even without a corresponding reduction in the overall incidence of AL, may therefore translate into fewer of these severe downstream consequences, independent of whether it changes the underlying leak rate.

Despite a reduction in clinical AL, no significant differences were observed in other outcomes, including overall and radiological AL, reoperations, mortality, postoperative infections, operative blood loss, length of hospital stay, and operative time. Merad et al. reported an exceptionally high reoperation rate, contributing to 64.8% of the weight in the analysis of this outcome [13]. They noted that 70% of these reoperations were due to causes unrelated to AL, such as local or generalized peritonitis, hemorrhage, wound disruption, obstruction, and postoperative acute cholecystitis. In contrast, other studies predominantly attributed reoperations to AL. A leave-one-out sensitivity analysis excluding Merad et al. shifted the pooled reoperation estimate from OR 0.63 (95% CI 0.37-1.08) to OR 0.41 (95% CI 0.17-1.01), suggesting that this study's disproportionate weight and high proportion of AL-unrelated reoperations may have attenuated the apparent effect of OMP on this outcome, though the revised interval still did not reach statistical significance.

This study has several limitations. First, four of the included studies were observational, with most being retrospective cohorts, potentially introducing selection bias and impacting the results. Second, several outcomes exhibited significant heterogeneity. Leave-one-out sensitivity analyses showed that the pooled estimates for overall AL, length of hospital stay, postoperative infection, and reoperation were each sensitive to the removal of a single study (Ozben et al. [9], Agnifili et al. [12], Nasiri et al. [14], and Merad et al. [13], respectively), while estimates for overall mortality, mortality due to abdominal complications, operative blood loss, and operative time remained materially unchanged on single-study exclusion. Third, the practical application of these findings may be influenced by variability in anastomotic closure techniques, stapled versus hand-sewn, as well as differences in the types of malignant and benign diseases and the specific anatomical locations involved in the colon and rectum. Fourth, planned subgroup analyses by study design (RCTs versus observational studies) and anastomotic technique (stapled versus hand-sewn) could not be performed due to the limited number of studies available within each subgroup, precluding a formal assessment of whether study design or anastomotic technique modified the pooled estimates. Finally, we did not extract data on the use of diverting stomas across the included studies. Since fecal diversion may alter the clinical presentation and detectability of an AL, its distribution between the OMP and n-OMP groups represents an unmeasured potential confounder of the clinical AL findings in this analysis.

## Conclusions

In conclusion, this systematic review and meta-analysis found no significant reduction in overall or radiological AL with OMP compared to n-OMP in colorectal surgery. However, clinical AL was significantly lower in the OMP group, a finding that should be interpreted with caution, as it rested on only three studies with few events (8/456 vs 24/487) and considerable practical uncertainty. This dissociation suggests that OMP may not prevent an anastomosis from leaking so much as limit the clinical severity of a leak once it occurs. No significant differences were observed in reoperation rates, mortality, postoperative infections, operative blood loss, length of hospital stay, or operative time. Given the discrepancies in the literature, the inclusion of observational studies, and the high heterogeneity observed for several outcomes, further well-designed RCTs are needed, particularly ones focused on low colorectal and pelvic anastomoses, where baseline leak risk and the theoretical benefit of omental reinforcement are greatest, to confirm these findings and clarify the role of OMP in colorectal anastomosis.
